# Characterization of Functional TRPV1 Channels in the Sarcoplasmic Reticulum of Mouse Skeletal Muscle

**DOI:** 10.1371/journal.pone.0058673

**Published:** 2013-03-11

**Authors:** Sabine Lotteau, Sylvie Ducreux, Caroline Romestaing, Claude Legrand, Fabien Van Coppenolle

**Affiliations:** 1 Université Lyon 1, Centre National de la Recherche Scientifique UMR 5534, Centre de Génétique et de Physiologie Moléculaire et Cellulaire, Villeurbanne, France; 2 Université de Lyon, Lyon, France; 3 INSERM U1060-CarMeN-“Equipe 5”, Lyon, France; 4 Université de Lyon 1, UMR 5023 Ecologie des Hydrosystèmes Naturels et Anthropisés, ENTPE, CNRS, Villeurbanne, France; Tohoku University, Japan

## Abstract

TRPV1 represents a non-selective cation channel activated by capsaicin, acidosis and high temperature. In the central nervous system where TRPV1 is highly expressed, its physiological role in nociception is clearly identified. In skeletal muscle, TRPV1 appears implicated in energy metabolism and exercise endurance. However, how as a Ca^2+^ channel, it contributes to intracellular calcium concentration ([Ca^2+^]_i_) maintenance and muscle contraction remains unknown. Here, as in rats, we report that TRPV1 is functionally expressed in mouse skeletal muscle. In contrast to earlier reports, our analysis show TRPV1 presence only at the sarcoplasmic reticulum (SR) membrane (preferably at the longitudinal part) in the proximity of SERCA1 pumps. Using intracellular Ca^2+^ imaging, we directly accessed to the channel functionality in intact FDB mouse fibers. Capsaicin and resiniferatoxin, both agonists as well as high temperature (45°C) elicited an increase in [Ca^2+^]_i_. TRPV1-inhibition by capsazepine resulted in a strong inhibition of TRPV1-mediated functional responses and abolished channel activation. Blocking the SR release (with ryanodine or dantrolene) led to a reduced capsaicin-induced Ca^2+^ elevation suggesting that TRPV1 may participate to a secondary SR Ca^2+^ liberation of greater amplitude. In conclusion, our experiments point out that TRPV1 is a functional SR Ca^2+^ leak channel and may crosstalk with RyR1 in adult mouse muscle fibers.

## Introduction

Muscle contraction is one leading mechanism where Ca^2+^ handling takes full extent and needs to be finely orchestrated to produce the appropriate signal encoded in space, amplitude and frequency. In a skeletal muscle cell, apart from the core EC (excitation-contraction) coupling machinery, DHPRs (dihydropyridine receptors) embedded in T-tubule membrane and RyRs (ryanodine receptors) in the sarcoplasmic reticulum (SR) membrane, and from the extrusion SERCA (sarco(endo)plasmic reticulum calcium ATPase) pumps, various other elements are recruited to perform this task. Among these latter, several members of the transient receptor potential (TRP) ion channel family were recently found expressed in mouse skeletal muscle [1]. Each element of the “Ca^2+^ tool kit”, submitted to internal and external messages, must communicate with each other and act in concert to maintain Ca^2+^ homeostasis. It is not surprising that any disturbance in the Ca^2+^ machinery can affect the well-being of muscle cell and lead to various muscle disorders from minor defect to severe myopathies [2]. For instance, mutations in human RyR1 are linked to malignant hyperthermia (MH) and central core disease (CCD) [35] and more recently some members of the vanilloid TRP subfamily have been associated with several hereditary diseases, i.e. mutations in TRPV4 linked to Charcot-Marie-Tooth disease type 2C [6], [7] and spinal muscular atrophies [8], [9] or else putative role of TRPV2 in Duchenne muscular dystrophy [10].

In most cell types, sarco/endoplasmic (SR/ER) reticulum is the largest intracellular Ca^2+^ reservoir. A number of works have revealed that the fine tuning of SR/ER Ca^2+^ homeostasis is the result of a balance between Ca^2+^ uptake by SERCA pumps and Ca^2+^ release through Ca^2+^ leak channels [11], [12]. Although SR Ca^2+^ leak mechanisms are still poorly understood, several members of the TRP family seem to be functionally important Ca^2+^ leak channels.

The aim of the present study was to explore the functionality and the subcellular localization of TRPV1 in the skeletal muscle. TRPV1 (formally known as VR1 for vanilloid receptor 1) is a putative six-transmembrane domain protein with a pore region between segments five and six and cytoplasmic N and C termini [13]. TRPV1 is a homotetrameric non-selective cation channel. It was first discovered as receptor for capsaicin, the pungent ingredient of chili pepper, in rat dorsal root ganglia [14]. Lately, TRPV1 was found in the sarcoplasmic reticulum of rodent skeletal muscle, and is likely responsible for release of Ca^2+^ into the cell [15]. By measuring contraction of rat skinned fibers, Xin and colleagues indirectly proved that TRPV1 is open and leaks already under relaxing conditions and that capsaicin activation enhances that leakage from the SR. In line with this finding, Cavuoto and colleagues [16] revealed that TRPV1 is also expressed in human skeletal muscle. A recent study has showed that TRPV1 activation by 4 months-administration of capsaicin could improve endurance capacity and energy metabolism in mice [17] underlying TRPV1 importance in skeletal muscle function. This capsaicin-induced TRPV1 activation is proposed to improve mitochondrial biogenesis and ATP production through a Ca^2+^-dependent PGC-1α (peroxisome proliferator-activated receptor-γ coactivator-1α) regulation. However, how TRPV1 directly impacts on the muscle Ca^2+^-homeostasis to influence the involved signaling pathways remains to be clarified.

As in rats, we show in this report that TRPV1 is expressed as a functional Ca^2+^ channel in mice. By isolating single fibers from mouse flexor digitorum brevis (FDB) muscle, we confirmed the expression of TRPV1 in adult skeletal muscle cells. Previous reports have let the question of TRPV1 plasma membrane expression with no clear answer [15]. The present study demonstrates that TRPV1 is present at the SR membrane but absent from the sarcolemme. Using intracellular Ca^2+^ measurements on intact fibers, we bring a critical extension in the TRPV1 knowledge by being the first to directly illustrate the Ca^2+^ mobilization caused by its activation in skeletal muscle. In particular, our work allows a better appreciation of the mechanistic involvement of TRPV1 in Ca^2^+ handling since our results are in favor of a functional TRPV1/RYR1 crosstalk. Since Ca^2+^ leakage can underlie muscle weakness and impaired muscle function [12], [18] our results provide new insight into SR Ca^2+^ release mechanism and suggest the implication of TRPV1 in the dysfunction of muscle SR Ca^2+^ release.

## Methods

### Ethics Statement

All experiments were performed in accordance with the guidelines of the French Ministry of Agriculture (87/848) and of the European Community (86/609/EEC). They were approved by the local animal ethic committee of Rhone-Alpes, approval number 692660602.

### Materials

Capsaicin, capsazepine, thapsigargin, CPA, type 1 collagenase, anti-protease cocktail and halothane were from Sigma-Aldrich. Resiniferatoxin was from Santa Cruz. Fluo4-AM and FM 1–43 were purchased from Molecular probes and Calbiochem respectively. Ryanodine was from Tocris. Nitrocellulose membrane was from BioRad. All other chemicals were of highest available grade.

### Animals

All experiments were performed using 4- to 8-weeks-old male OF1 mice. Mice were purchased from Charles River Laboratories.

### Western Blot Analysis

Five male mice were sacrificed by cervical dislocation, tissues (muscles, brain) were quickly removed and freshly used or frozen (−80°C) for later experiment. TRPV1 and SERCA expression levels were observed in tissue homogenate or microsomal preparation. For microsomal preparation, hind muscles were removed and all subsequent steps were performed at 4°C. The isolation of SR membrane fractions was obtained as previously described [19].

Tissues were homogenized in ice-cold buffer (Tris 5 mM, EGTA 2 mM, pH 7.4) supplemented with anti-protease cocktail. Homogenates were centrifuged at 3000 rpm for 10 min at 4°C. Supernatants were then centrifuged at 13000 rpm for 30 min at 4°C. Final pellets were resuspended in homogenization buffer.

SDS/PAGE and Western blot analysis were performed as following: proteins were separated on a SDS-PAGE 7.5% gel and transferred onto nitrocellulose membrane. The blots were blocked with 5% milk for 1 h and incubated with anti-TRPV1 (1∶200; Santa Cruz) or anti-SERCA1 (1∶2,500; Bioreagents) overnight at 4°C. The blots were then incubated with secondary antibodies anti-rabbit or anti-mouse HRP conjugated (1∶10,000; Sigma) for 1 h at RT, developed in enhanced chemiluminescence solution (ECL Plus; Amersham) for 5 min.

### Isolation of Muscle Fibers

Most of the experiments were performed on single skeletal fibers isolated from the flexor digitorum brevis muscles of wild-type (OF1) male mice. In brief, mice were killed by cervical dislocation. Muscles were removed and treated with type 1 collagenase for 45–60 min at 37°C in the presence of Tyrode as external solution. Single fibers were then obtained by triturating muscles within the experimental chamber. Cells were mounted into a glass bottom dish. Fibers were bathed in the presence of Fluo-4 AM (5 µM) during 30 min. Cells were then washed with Tyrode. Tyrode solution contained (in mM): 140 NaCl, 5 KCl, 2.5 CaCl2, 2 MgCl2 and 10 HEPES and 5.5 glucose adjusted to pH 7.4 with NaOH.

### Immunostaining

Single skeletal fibers isolated from the flexor digitorum brevis muscles of wild-type male mice were fixated and permeabilized by methanol (−20°C during 7 min) and rehydrated 1 h in PBS (Phosphate Buffered Saline 1X, Sigma). SERCA 1 antibodies were obtained from Bio reagent (1∶500), RyR 1 antibodies from Sigma (1/1000) and TRPV1 antibodies from Alomone (1∶100) or from Abnova (1∶1000). As secondary antibodies, goat anti-mouse or goat anti-rabbit from Invitrogen conjugated to fluorophores Alexa 488 or Cy3. Fluorescence of immunostaining was measured on a Zeiss LSM 5 Exciter laser scanning confocal microscope.

### Labeling of Mouse Isolated Muscle Fibers with FM1–43 Dye

Single fibers, isolated as detailed above, were mounted into thin bottom plastic dishes (Ibidi, Biovalley) coated with 1 µg/ml laminin. Images were obtained from intact or saponin-permeabilized fibers. To permeabilize the surface membrane, cells were treated with a modified relaxing solution (125 mM K-glutamate, 10 mM HEPES, 1 mM EGTA, 6 mM MgCl2, 5 mM Na2-ATP, 10 mM Na-phosphocreatine, 10 mM glucose, 0.13 mM CaCl2) containing 0.002% saponin as described before [20]. Permeabilization was monitored by the addition of FM1–43 into the solution. Control fibers were incubated in 10 µM FM1–43 alone or together with 100 µM capsaicin for 5 min, and washed in relaxing solution to remove free FM1–43 as previously described (Meyers et al. 2003).

### Confocal Ca^2+^ Imaging and Image Analysis

Unless otherwise specified, imaging was achieved on a Zeiss LSM 5 Exciter laser scanning confocal microscope. The microscope was equipped with a 63x oil immersion objective (NA = 1.4). Fluo-4 and FM1–43 were excited with 488 nm argon laser. Their respective emitted fluorescent light were measured at wavelengths >505 nm. Since calcium responses to TRPV1 agonists revealed slow kinetic, images (512/512 pixels) were taken with a 5 or 15 seconds interval. Fluorescence of regions of interest were normalized to baseline fluorescence (F 0 [x]). Experiments were performed at room temperature.

### Data Analysis

Results were expressed as the means ± S.E.M. Statistical analysis was performed using Student's t test (differences considered significant when p was <0.05) and Origin 5 software (Microcal Software Inc.).

## Results

### TRPV1 Expression and Localization in Mouse Skeletal Muscle

**Figure 1:**
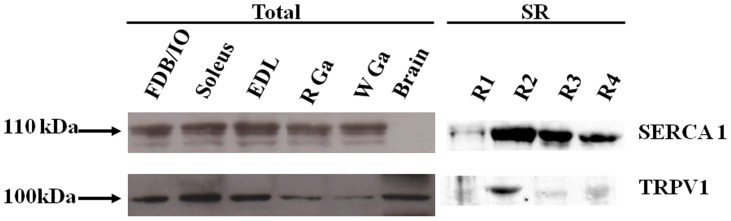
Expression of TRPV1 and SERCA1 in skeletal muscle. Left panel: Western blot analysis of total protein extracts (25 µg) with anti-SERCA1 (top panel) and anti-TRPV1 antibodies (low panel) from the following tissues: flexus digitorum brevis/interosseal (FDB/IO), soleus, extensor digitorum longus (EDL), red and white gastrocnemius (R Ga and W Ga) and brain. Right panel: Localization of TRPV1 in sarcotubular membrane fractions (R1, fraction enriched in light SR; R2, fraction enriched in longitudinal SR; R3, fraction containing a mixture of longitudinal SR and terminal cisternae; R4, fraction enriched in terminal cisternae): 30 µg of protein from each fraction was separated on SDS/PAGE.

**Figure 2:**
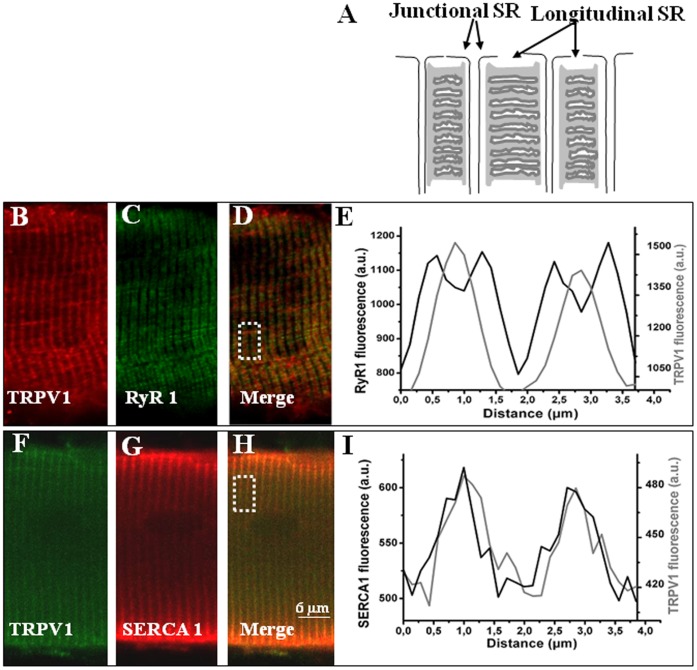
Localization of TRPV1, RyR1 and SERCA1 in mouse FDB fibers. A: Schematic organization of the sarcoplasmic reticulum (SR) in a skeletal muscle fiber. Confocal images of double immunofluorescence labeling of TRPV1 (B) and RyR1 (C) or TRPV1 (F) and SERCA1 (G). E, I: average intensity profiles from the dotted rectangle region in the next corresponding images. In isolated fibers, RyR1 shows localization in the junctional part of the SR (C) while SERCA1 appears in the longitudinal part of the SR (G). In the merged images, TRPV1 does not colocalize with RyR1 (D) while colocalization with SERCA1 staining is visible in H. Results are from at least 4 independent fibers preparations (n>10).

Skeletal muscle TRPV1 expression was determined by Western blot analysis of total extracts from different muscle types or of SR membranes isolated from wild-type mice (Fig. 1). TRPV1 protein was abundant in the different muscles tested, FDB and interosseal, EDL, soleus, white and red gastrocnemius (Fig. 1, left). To date, the sarcoplasmic reticulum is composed by the junctional part (near the t-tubules) and longitudinal part (Fig. 2A). To determine more precisely TRPV1 location, microsome fractions were obtained from predominantly white portions of the hind muscles. Fractions were as following: R1, fraction enriched in light SR; R2, fraction enriched in longitudinal SR; R3, fraction containing a mixture of longitudinal SR and terminal cisternae; R4, fraction enriched in terminal cisternae. SERCA1 is found in R2 to R4 fractions (with a higher content in fractions R2) [19] while TRPV1 appeared in longitudinal preparation (R2) (Fig. 1, right).

To assess optimally the physiological TRPV1-function in adult muscle, we used isolated muscle fibers from the mouse FDB as experimental model. We began with defining the cellular localization of the TRPV1 protein. Using immunocytochemistry, we also found TRPV1 expressed in the sarcoplasmic reticulum but not detected at the plasma membrane (Fig. 2B and 2F). To check the position of TRPV1 within the SR, we realized co-immunolocalization experiments with specific markers: RyR1 and SERCA1, respectively positioned in the junctional part and in the longitudinal part of the SR (Fig. 2C and 2G).

Our team had previously developed techniques to localize RyR1 in the same cell model [21] and illustrate the pattern expression of another TRP channel, TRPC1, with a longitudinal SR localization [22]. Here, RyR1 was observed at its predicted triadic position (Fig. 2C). As illustrated on the expression profile (Fig. 2E), the RyR1 labeling showed 2 µm- alternation of double 1 µm-spaced peaks in accordance with the well-known triadic localization of RyR1 in skeletal muscle cells. Regarding TRPV1, we only observed single peaks repeated every 2 µm. Similar labeling and localization were obtained using another anti-TRPV1 antibody (see Fig. S1). These peaks did not overlay with those of RyR1 meaning no colocalization of the two respective proteins (Fig. 2D). When we secondly tested immunolocalization between SERCA1 and TRPV1, SERCA1 localization in the SR longitudinal part (Fig. 2G) was translated by cycles of single 2 µm-distant peaks on its expression profile (Fig. 2I). TRPV1 and SERCA1 expression profiles were superimposed attesting their presences in the SR longitudinal part.

**Figure 3:**
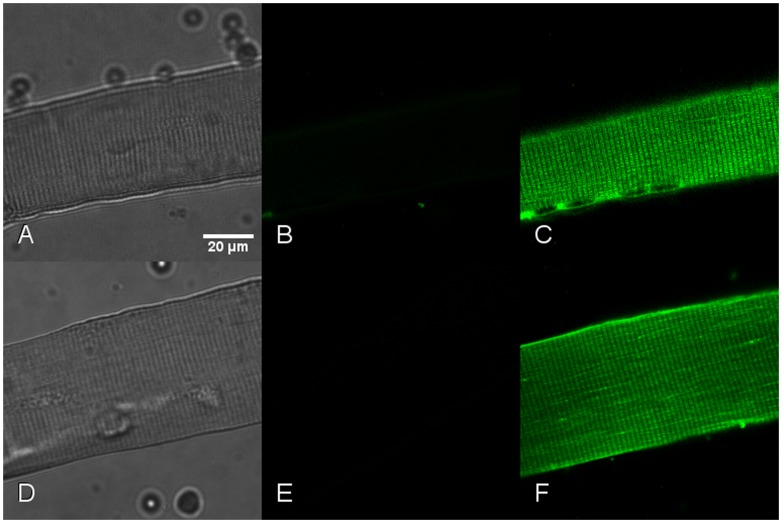
FM1–43 labeling in intact and permeabilized FDB isolated muscle fibers. In the top panel (A; B; C) cells were incubated in relaxing solution; in the low panel (D; E; F), cells in relaxing solution containing capsaicin (100 µM). Photomicrographs in transmitted light (A, D) or FM1–43 fluorescence in cells before (B; E) or after (C; F) saponin-permeabilization. In intact cells (B; E), FM1–43 fails to enter the FDB fiber, while once the surface membrane is disrupted, the dye enters into the cytoplasm and labels SR membranes (C; F). Results are from at least 2 independent fibers preparations (n>3).

Since TRPV1 has been previously identified both at the plasmalemma and at the endoplasmic membrane in other tissues [23], [24], we wanted to ensure the true absence at the plasma membrane site observed above. To this end, we next tested the TRPV1 permeability to the styryl pyridinium dye FM 1–43. FM 1–43 is a cationic dye used to measure vesicle recycling and its fluorescence increases after incorporation into membrane. FM 1–43 is able to cross the plasma membrane using TRPV1 channels when existing [25]. Thus, external application of capsaicin is a method to assess the presence of functional isoforms of TRPV1 at the plasma membrane. In our experiments, to evaluate the FM 1–43 entry, intact or permeabilized cells were bathed with the dye (Fig. 3A–3D). In intact fibers, the FM 1–43 fluorescence was negative in basal conditions (Fig. 3B, relaxing solution); while damaged fibers where plasmalemmal membrane integrity is disrupted, were clearly fluorescent (data not shown). As the surface membrane was permeabilized by saponin-treatment, fluorescence first appeared at the periphery of the fiber, moved quickly into the cytoplasm and labeled SR membrane (Fig. 3C). When the same experiments were repeated in the presence of capsaicin, intact fibers remains unstained (Fig. 3E) while in permeabilized ones, the dye brightly labeled SR within 1 min (Fig. 3F). In accordance with the colocalization experiments, these data set TRPV1 expression only at the SR membrane.

### TRPV1 is Functional in Isolated Muscle Under Pharmacological Activation

**Figure 4:**
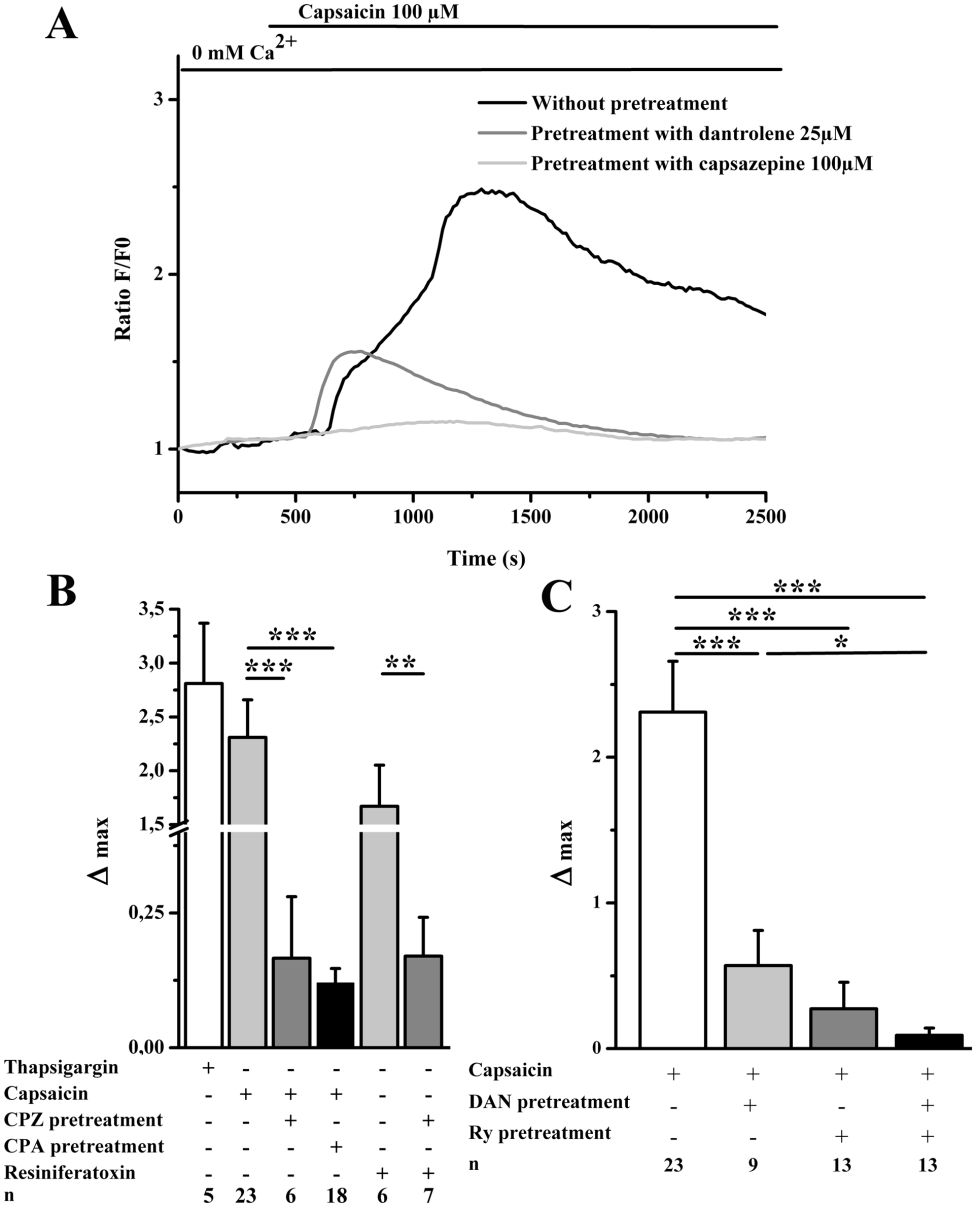
Calcium release induced by pharmacological activation of TRPV1 in FDB isolated fibers. (A) The traces show representative curve obtained after stimulation of single fibers with capsaicin alone (100 µM; black line) or in the presence of capsazepine (light grey line) or dantrolene (dark grey line). B; C: changes in fluorescent ratio F/F0 (peak– resting) induced by drugs as indicated in table above the graphs. Summary data showing fluorescent changes in cells induced by agonists, B: with or without antagonist pretreatment or else after SR depletion and C: with or without RyR inhibitors pretreatment. Capsazepine (CPZ, 100 µM), cyclopiazonic acid (CPA, 25 µM), dantrolene (DAN, 25 µM) and ryanodine (Ry, 80 µM) were added 20 min prior to agonist treatment. Resiniferatoxin and thapsigargin were used at 10 µM and 1 µM respectively. Results are expressed as means ± S.E.M. of the indicated number of experiments from at least 4 independent fibers preparations. T-tests were performed by paired samples: *p<0.05, **p<0.002 and ***p<0.0002.

Prior to the present study, TRPV1 presence in mouse skeletal muscle was already evident. However, the channel’s characterization regarding Ca^2+^-mobilization was never attempted in this model but shortly explored in cultured myotubes [17]. Here, to investigate a possible involvement of TRPV1 in the muscle Ca^2+^ homeostasis, we next carried out Fluo-4 Ca^2+^ measurements on isolated FDB fibers. To avoid capacitive Ca^2+^ entry, all experiments were done in a Ca^2+^-free medium. We first examined the status of intracellular Ca^2+^ stores by adding SERCA inhibitor thapsigargin (1 µM). The peak of fluorescence related to [Ca^2+^]_i_ increase obtained in our experimental condition was 2.81±0.56 (Δmax = change in fluorescent ratio F/F0 (peak– resting); Fig. 4B). Even if thapsigargin is a TRPV1 blocker, it has higher affinity for SERCAs than for TRPV1 and would block this latter only if added in pretreatment and at higher concentrations [26], [27]. Fig. 4A illustrates a representative single FDB [Ca^2+^]_i_ measurement experiment: addition of the agonist capsaicin resulted in an increase in the [Ca^2+^]_i_ (Δmax = 2.31±0.35). Alternatively to capsaicin, experiments were also performed using another ultrapotent TRPV1 agonist, resiniferatoxin (10 µM) and provided similar results (Δmax = 1.67±0.38; Fig. 4B). The maximal capsaicin-induced Ca^2+^ release and resiniferatoxin-induced Ca^2+^ release were significantly reduced by a pretreatment with capsazepine (CPZ, 100 µM), a TRPV1 antagonist (Δmax = 0.17±0.11 and Δmax = 0.17±0.07 respectively; Fig. 4B). The capsaicin-evoked Ca^2+^ release after SR depletion (20 min-pretreament with CPA, 25 µM) was almost null (Δmax = 0.12±0.027; Fig. 4B) showing that TRPV1 activation mostly recruits SR Ca^2+^. These results clearly indicate TRPV1 are functional channels in the SR that might act as Ca^2+^ leak channels in mouse skeletal muscle cells. It is noticeable that the concentration of capscaicin necessary to induce Ca^2+^ release was relatively high (100 µM) in our experimental conditions. Our choice was in agreement with the work of Gallego-Sandin and coworkers [24] showing that the ER isoform of TRPV1 has a smaller sensitivity to capsaicin than the plasma membrane channels in dorsal ganglions neurons.

Note also that the SR Ca^2+^ response to capsaicin occurred in two phases (Fig. 4A). In order to determine the provenance of the Ca^2+^ released, we inhibited the SR release from RyR1 by blocking the receptor with ryanodine (80 µM). In this condition, capsaicin failed to induce a Ca^2+^ release with the same large amplitude (Δmax = 0.27±0.18, Fig. 4C). Consistently, we also obtained an inhibition in the capsaicin response when we applied dantrolene (DAN, 25 µM), an inhibitor of SR Ca^2+^ release in skeletal muscle cells [28] (Δmax = 0.57±0.24; Fig. 4C).Dantrolene inhibition was slightly less (but not significantly) efficient than ryanodine’s one, probably because the binding sites for these two drugs are pharmacologically distinct [29]. When cells were then submitted to the double pretreatment (dantrolene plus ryanodine), the capsaicin-induced Ca^2+^ release was even more dramatically reduced (Δmax = 0.092±0.049), indicating that the contribution of TRPV1 to the release is minimal but large enough to initiate a greater SR response. We proposed that under capsaicin activation, TRPV1 would release Ca^2+^ from the SR to the cytoplasm. In a second time, the SR Ca^2+^ release would be then amplified by the activation of RyR1 and other not yet characterized SR Ca^2+^ leak channels.

### Physiological Activation of TRPV1

**Figure 5:**
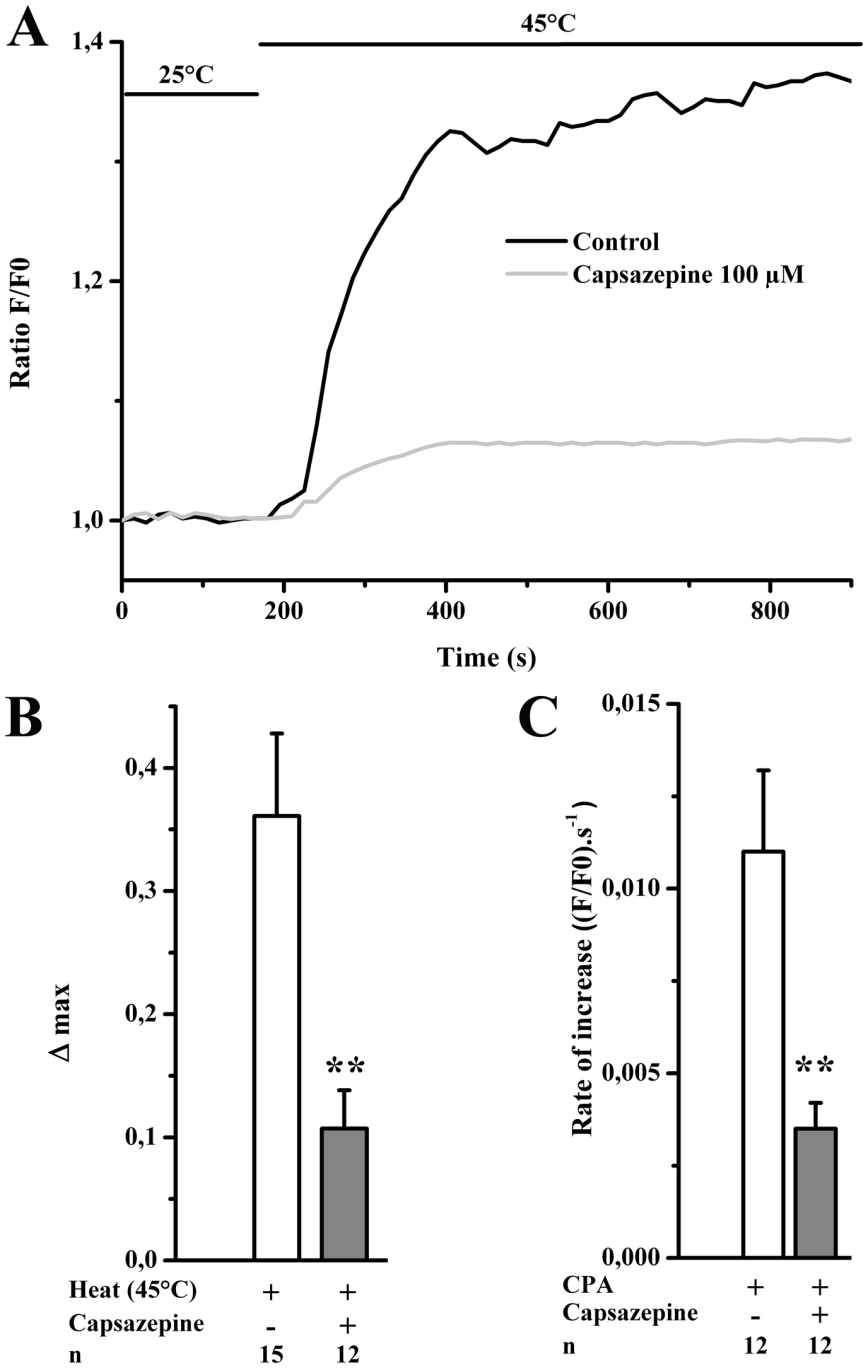
Calcium release induced by physiological activation of TRPV1 in FDB isolated fibers. (A) Time course of cytosolic Ca^2+^ concentration in Fluo-4 loaded cells in response to heat. The temperature of external Ca^2+^-free solution was increased from 25°C to 45°C (as depicted by horizontal bars on the top). Experiments were performed with or without capsazepine pre-treatment (100 µM during 25 min). (B) Cumulative data of the heat effect on SR Ca^2+^ release in control conditions or after capsazepine pre-treatment. (C) Cumulative data of the rate of SR Ca^2+^ release evoked by CPA (25 µM) in control conditions and after TRPV1 inhibition by capsazepine. Results are expressed as means ± S.E.M. T-tests were performed by paired samples: **p<0.06.

In order to verify that our pharmacological approach was relevant to muscle physiology, we ascertained that TRPV1 activation occurs under physiological activation using temperature elevation [14]. Briefly, once [Ca^2+^]_i_ was stable at room temperature (25°C ), hot extracellular medium (Tyrode; 45°C) was perfused. Figures 5A and 5B illustrate the effect of heat on SR Ca^2+^ release, i.e. an increase in [Ca^2+^]_i_ (Δmax = 0.37±0.07; Fig. 5B). This temperature effect remained insignificant in capsazepine (100 µM; 25 min) pre-treated cells (0.11±0.03). Thus, these results indicated that TRPV1 might have a physiological relevance. The next question was to assess whether TRPV1, located in the SR membrane, is already physiologically activated at room temperature (25°C) and acts as a functional SR Ca^2+^ leak channel in skeletal muscle cells. In resting conditions, [Ca^2+^]_SR_ is an equilibrium between Ca^2+^ release through passive Ca^2+^ leak channels and Ca^2+^ reuptake by SERCA pumps. To reveal the SR Ca^2+^ leak, SERCA pumps were inihibited by CPA and the rate of CPA-induced Ca^2+^ release was calculated. The slope of the [Ca^2+^]_i_ increase was significantly reduced under TRPV1 inhibition by capsazepine pre-treatment (0.0035±0.0007) as compared to control conditions (0.011±0.0022; Fig. 5C). Altogether, these data demonstrate that TRPV1 is physiologically activated at room temperature and participates to SR Ca^2+^ release in fully differentiated mouse skeletal muscle cells.

## Discussion

In skeletal muscle tissue, almost 10 different TRP channels have been detected [30]. Nonetheless, we are still in the early phases to understand their impact in skeletal muscle function. Originally, TRPV1 was cloned as a capsaicin receptor from neuronal cells of dorsal root ganglia but was recently found in a few non-neuronal tissues, including skeletal muscle [15], [16], [31]. These preceding observations encourage us to first determine whether TRPV1 is express in either slow or fast-twitch muscle type and we found TRPV1 presence in both sub-types (Fig. 1). Previous works based on skinned fibers or cultured myotubes have reported TRPV1 expression in both SR and plasma membrane [15], [17]. In particular, TRPV1/SERCA1 co-localization has already been showed in rat fibers by confocal and electronic microscopy [15]. Our initial aim was to verify this fact in our experimental model (mouse FDB), this verification by confocal microscopy led us to take a particular attention at TRPV1 sarcolemmal expression. Using adult mouse isolated muscle fibers and microsomal preparation (Fig. 1 & 2), our data confirmed the localization of TRPV1 in the longitudinal part of the SR but in contrast revealed no labeling at the plasma membrane. Other evidence comes from our FM1–43 entry experiments, a dye widely used to measure uptake by endocytosis. Meyers and colleagues [25] have shown that FM1–43 rapidly entered in cells expressing TRPV1 after stimulation by agonists like capsaicin. When we used this assay to show up the putative plasmalemmal localization of TRPV1, no increase in FM1–43 fluorescence was detected in intact fibers after capsaicin addition (Fig. 3). We thus conclude that TRPV1 is only present in the SR of fully differentiated mouse skeletal muscle cells. It would be interesting to verify if under certain physiological conditions, TRPV1 could translocate from intracellular pools to the membrane similarly to TRPV2 [32].

We then check the TRPV1 functionality in mouse skeletal muscle cells, carrying out experiments in the absence of external Ca^2+^ to avoid capacitive Ca^2+^ entry. The TRPV1 implication in the SR Ca^2+^ leak has been previously revealed in skeletal muscle but the results were obtained from skinned fibers [15]. Under caffeine induction, Xin *et al.* performedcontraction measurements and interpreted developed tension as a Ca^2+^-indicator. Thereby, they successfully demontrated TRPV1 role in muscle contraction and in SR Ca^2+^ leakage. While using similar tools (capsaicin, dantrolene, capsazepine) and others (resiniferatoxin, CPA, thapsigargin, ryanodine, temperature), we straight focalized on Ca^2+^-transients to better understand how TRPV1 contributes to the Ca^2+^ mobilization *per se*. In the present report, we used intact fibers not to bias our results with an artificial intracellular environment. Due to their lipophilic properties, capsaicin and its analogues, such as resiniferatoxin, can cross the plasma membrane and may activate TRPV1 channels located in the SR. This journey might explain the relatively long delay observed between agonist application and the increase in Ca^2+^ release (157±41.83 s). The concentration of capsaicin used to activate the SR-located TRPV1 in our cell model is similar to the one of Gallego-Sandin and coworkers employed to stimulate TRPV1 in the ER of dorsal root ganglion neurons [24]. Our results are the first to straight show that TRPV1 is a functional SR Ca^2+^ channel in adult muscle cells. In this report, we observed a lowered increase in [Ca^2+^]_i_ after TRPV1 activation by heat as compared to the one obtained by pharmacological components (capsaicin and resiniferatoxin). These results could be due to the fact that: (i) heat sensibility and capsaicin binding sites are different [14], capsaicin binds to tyrosine 511 between the S3 and S4 segments, while the C-terminus domain (V686–W752) renders TRPV1 sensitive to heat; (ii) heat and capsaicin induce a Ca^2+^-dependent desensitization of TRPV1 [33]. These Ca^2+^-dependent mechanisms are still incompletely understood especially for the ER/SR located TRPV1 channels. Interestingly, the SR Ca^2+^ leak after capsaicin or resiniferatoxin perfusion occurs in two phases. In a first step, Ca^2+^would be released through TRPV1 after its activation. The second step would be the subsequent RyR1 Ca^2+^release due to low increased Ca^2+^ levels. Several works especially on RyR1 incorporated into lipid bilayers have demonstrated RyR1 properties of CICR (calcium-induced calcium release) activity but the physiological significance of CICR remains a controversial issue in skeletal muscle [34]. Here, this step could not be accurately related to a CICR activity of ryanodine receptors for spatio-temporal reasons: (i) RyR1 and TRPV1 may be physically too far away from each other (illustrated by the Fig. 2) and (ii) the response kinetic may be too slow. As opposite, our results can be an argument for CICR existence and we could speculate that the long distance between RYR1 and TRPV1 only affect the kinetics parameters. Nevertheless, the fact that the second step was abolished with pre-treatment of ryanodine,and/or dantrolene during capsaicin response favors the correspondence between the second phase and RyR1 activation but does not exclude other Ca^2+^ leak channels contribution.

When considering the localization of TRPV1 within skeletal myofibril, our data show that the channel is present only at longitudinal part of the reticulum (Fig. 2). In the resting state, the Ca^2+^ content of the SR reflects a balance between the active uptake of Ca^2+^ by SERCA and passive efflux through leak channels. Thus, the Ca^2+^ leak which could occur through TRPV1 is certainly unnoticed because probably buffered by the vicinity with SERCA1 pumps. In this study, we directly show, in our cell model, that TRPV1 is activated at room temperature. As mentioned earlier, SERCA pump inhibition by CPA reveals SR Ca^2+^ leak and Ca^2+^ leak channels activities. Here, the slope of [Ca^2+^]_i_ increase due to CPA-induced Ca^2+^ release was reduced after TRPV1 inhibition by capsazepine. These data demonstrate that the SR-located TRPV1 of skeletal muscle cells and acts as a functional SR Ca^2+^ leak channel in resting conditions. Note that TRPV1 activation by agonist can lead to empty the SR-stores in the same extend allowed by blocking SERCA pumps (Ca^2+^ response to capsaicin, resiniferatoxin and thapsigargin were from the same amplitude). At a certain threshold, SERCA pumping would not be able to entirely counteract Ca^2+^ leak through TRPV1. This phenomenon observed in our experimental conditions could remain undetected when external and internal environments are clamped by the experimental system.

**Figure 6:**
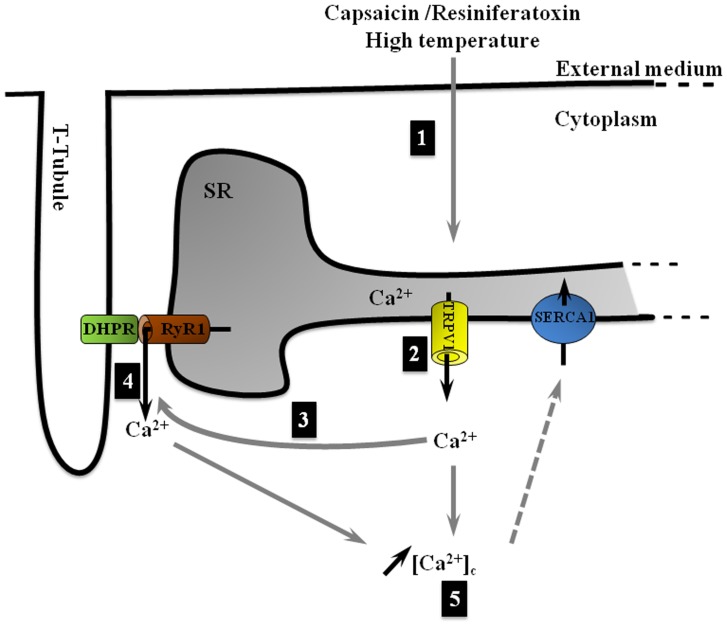
Schematic drawing of the possible TRPV1 contribution in the maintenance of Ca^2+^ muscle homeostasis. (1) External stimuli, such as capsaicin, resiniferatoxin or high temperature, activate TRPV1 channels located at the longitudinal SR. (2) TRPV1 channels releases Ca^2+^ from SR to cytoplasm. (3) This low increase in [Ca^2+^]_c_ activates RyR channels (4) that in return release Ca^2+^ into cytoplasm increasing [Ca^2+^]_c_ (5). In permanence, SERCA1 pumps continuously back Ca^2+^ from cytoplasm to SR and could mask TRPV1 activation in resting condition.

In addition, surrounding buffers such as calmodulin (CaM) could mask TRPV1 action. Indeed, CaM was reported to be involved in a Ca^2+^-dependent desensitization of TRPV1 in neurons [35]. We could imagine if TRPV1-CaM interaction also exists in muscle, once TRPV1 is activated, Ca^2+^ might leak through the SR to the cytoplasm and bind to CaM. At that time, CaM might desensitize TRPV1 and induce a decrease or a termination of Ca^2+^ release via these channels. In parallel with the calcium CaM-buffering capacities of skeletal muscle cells, it could explain the bell-shape response obtained after capsaicin or resiniferatoxin stimulation. Thus, the Ca^2+^ leak might become measurable only after capsaicin treatment or phenomenon which induces the recruitment of TRPV1 channels. Taken together the results of our study, we proposed a new concept of TRPV1 implication in the Ca^2+^ homeostasis (Fig. 6).

Other TRP family members have already been reported as functional channels in skeletal muscle. Among them, the Transient Receptor Potential Canonical type 1 (TRPC1) could operate as a SR Ca^2+^ leak channel in skeletal muscle [22]. Recently, it has been reported that TRPV4 could modulate Ca^2+^ influx and muscle fatigue [36] and is furthermore involved in skeletal dysplasia [37]. But unlike TRPV1 or TRPC1, TRPV4 is localized in the sarcolemma in mouse muscle [38]. Adding TRPV1, we lengthen the list of biologically efficient channels for the muscle physiology. In a recent work, TRPV1 activation by dietary capsaicin supplementation had revealed one of its roles in skeletal muscle function. Capsaicin diet improved exercise endurance and energy metabolism by increasing PGC-1α expression and mitochondrial biogenesis in a Ca^2+^-dependent mode [17]. In parallel, TRPV1-KO mice become over-weighted with age (observed for up to 14 months) as compared to controls [39]. In this conceptual picture, we should also remember that TRPV1 is activated by low pH [40], and also function as temperature sensors like [41]. TRPV1 is sensitive to heat (max activation at 45°C) [14]. In this work, we demonstrate that the SR-located TRPV1 of skeletal muscle cells is functional and activated by heat. These results are physiologically relevant since local temperature elevation can occur during muscle exercise or accompanied muscle fatigue. A basal Ca^2+^ leak from the SR to the cytosol may occur through TRPV1 in any of these particular conditions. We are also convinced that Ca^2+^ release via TRPV1 could be a crucial step for activation of RyR1 and that these effects could have clinical relevance. In future, next investigations will have to decorticate the role of TRPV1 in skeletal muscle function and to reveal which signals or mechanisms are dependent on specific activation of TRPV1 rather than another TRP family member.

## Supporting Information

Figure S1 **Localization of TRPV1 in mouse FDB fibers.** A and B: Confocal images of immunofluorescence labeling of TRPV1 using Abnova antibody (A; 1∶1000) or Alomone antibody (B; 1∶100). (C) average intensity profiles from the dotted rectangle region in the next corresponding images. In isolated fibers, TRPV1 display identical profiles with the 2 different antibodies used. The distances between each peak is 2 µM corresponding to a specific localization of TRPV1 within the longitudinal part of the SR. Results are from at least 4 independent fibers preparations (n>10).
(TIF)
