# Supplementary figures and images for: Characterization of Functional TRPV1 Channels in the Sarcoplasmic Reticulum of Mouse Skeletal Muscle
Source: PLoS One. 2013 Mar 11;8(3):e58673. doi: 10.1371/journal.pone.0058673 (PMC3594164; doi:10.1371/journal.pone.0058673)

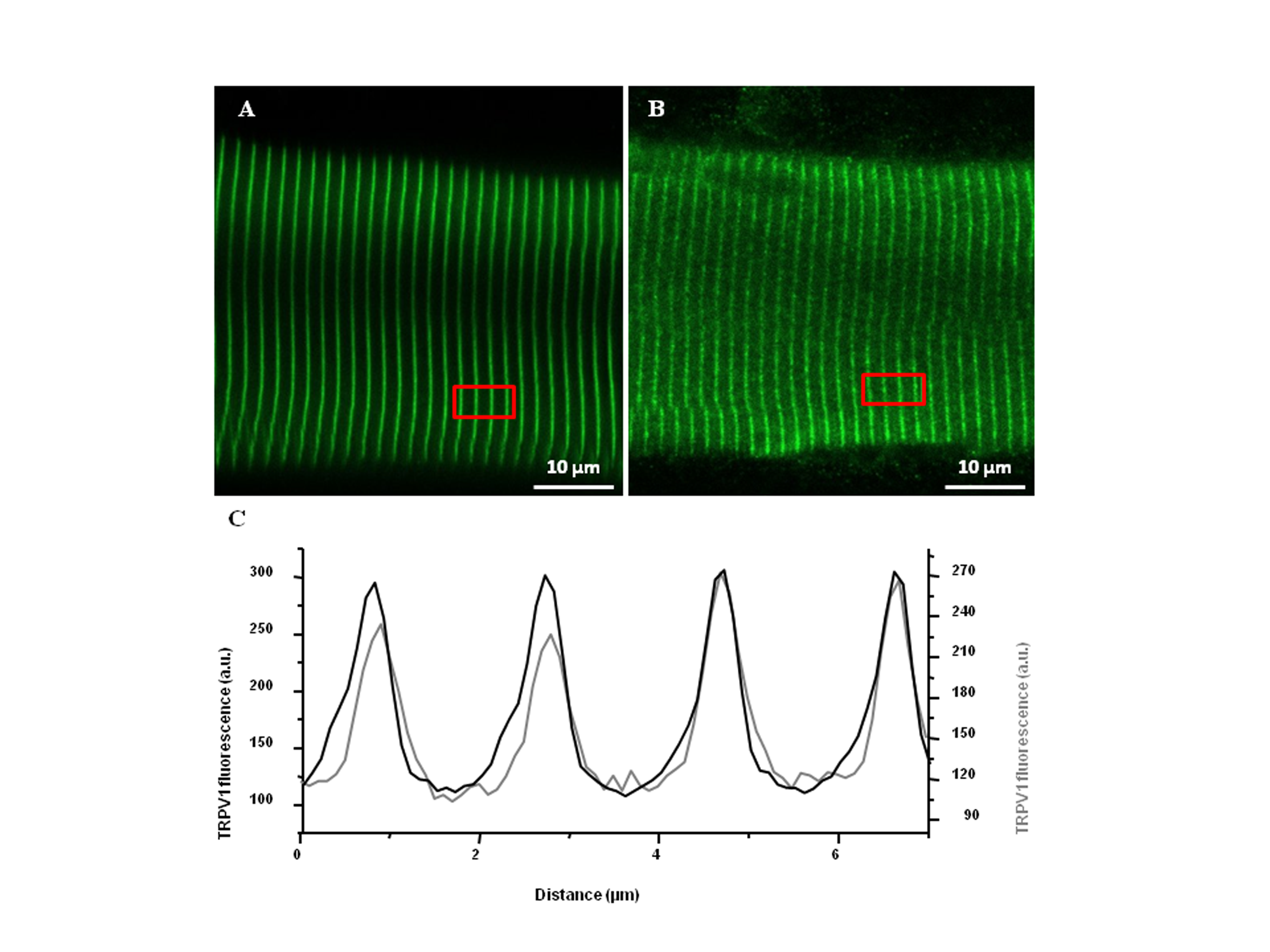

Supplement: Figure S1 — Localization of TRPV1 in mouse FDB fibers. A and B: Confocal images of immunofluorescence labeling of TRPV1 using Abnova antibody (A; 1∶1000) or Alomone antibody (B; 1∶100). (C) average intensity profiles from the dotted rectangle region in the next corresponding images. In isolated fibers, TRPV1 display identical profiles with the 2 different antibodies used. The distances between each peak is 2 µM corresponding to a specific localization of TRPV1 within the longitudinal part of the SR. Results are from at least 4 independent fibers preparations (n>10). (TIF) [file pone.0058673.s001.tif]
